# Addressing HIV criminalization: science confronts ignorance and bias

**DOI:** 10.1002/jia2.25163

**Published:** 2018-07-25

**Authors:** Kenneth H Mayer, Annette Sohn, Susan Kippax, Marlène Bras

**Affiliations:** ^1^ The Fenway Institute Fenway Health and Department of Medicine Beth Israel Deaconess Medical Center/Harvard Medical School Boston MA USA; ^2^ TREAT Asia/amfAR The Foundation for AIDS Research Bangkok Thailand; ^3^ Social Policy Research Centre University of New South Wales Sydney NSW Australia; ^4^ Journal of the International AIDS Society Geneva Switzerland

**Keywords:** Human rights, HIV, Criminalization

About 70 countries continue to criminalize non‐disclosure of HIV‐positive status, exposure to HIV and transmission of HIV without a scientific basis. No data support HIV criminalization as a supposed deterrent to protect innocent people from becoming HIV positive. On the contrary, criminalization of HIV transmission creates an unjust public health environment where individuals living with HIV may be fearful about disclosing their status, which may delay their own engagement in care. In a world where highly effective tools exist to enhance the lives of people living with HIV and to curtail HIV transmission through the ongoing use of effective antiretroviral medication, any disincentive to engage in care is undesirable from a public health and human rights standpoint.

When the first reports of what came to be known as the AIDS epidemic appeared in the early 1980s, concerns were raised about how the virus could be transmitted 1. Within the next several years, the aetiologic agent was elucidated and blood tests were available to determine who was, and was not, infected 2. These assays, coupled with meticulous epidemiologic investigations, showed that HIV was transmitted by intimate sexual contact and parenteral blood and blood product exposure. Subsequent studies helped refine the understanding of the relative infectiousness of different exposures and, very quickly, a hierarchy of potential sexual and parenteral transmission risks emerged 3.

Although HIV acquisition is a high‐consequence event, the relative efficiencies of HIV transmission are quite low. Furthermore, in the highly active antiretroviral therapy era, transmission rates are declining in communities where the majority of people living with HIV are on treatment and have suppressed viral loads. The likelihood of intentional HIV transmission is extremely rare since it requires premeditated intent of an HIV‐infected individual to transmit HIV, lack of virological suppression with effective treatment 4, and/or non‐consensual sex. Based on several decades of intense scrutiny by public health and criminal justice officials, such cases are rare, and usually can be addressed with existing laws and jurisprudence.

The *Journal of the International AIDS Society* is pleased to publish the “Expert consensus statement on the science of HIV in the context of criminal law” 5. We feel that this document, developed by 20 expert scientists from regions across the world, is an important step to combat the ignorance that underlies the continued existence of laws that criminalize HIV transmission.

As is explained in the consensus statement, specific laws focusing on HIV criminalization, and misuse of other laws despite the evidence against the likelihood of HIV transmission, reflect the perpetuation of ignorance, irrational fear and stigmatization, or a punitive intent directed towards people living with HIV – whether related to intolerance of key populations and behaviours, or other forms of social exclusionary thinking.

The consensus statement takes great pains to help orient a broad readership with how the data may best be interpreted. It is the hope of the Editors that this document will better inform readers about the reasons why criminalization will not help reduce transmission, but only fuel the epidemic. We therefore hope that governmental authorities will view this consensus statement as a resource to better understand the actual rather than the perceived risks posed by exposures to individuals living with HIV, and to create societies that encourage engagement and not fear.

## Competing interests

None.

## Authors’ contributions

All authors have contributed to the preparation of the manuscript, read and approved the final draft.
